# Influence of core self-evaluation on social anxiety in college students with financial difficulties: mediation of self-acceptance

**DOI:** 10.3389/fpsyg.2025.1607544

**Published:** 2025-11-20

**Authors:** Yu-E Chen, Li-Ying Lai

**Affiliations:** 1 Mental Health Center, Sanming University, Sanming, China; 2 School of Foreign Languages, Sanming University, Sanming, China

**Keywords:** college students, financial difficulties, core self-evaluation, social anxiety, self-acceptance

## Abstract

This study investigated the mediating role of self-acceptance in the relationship between core self-evaluations and social anxiety among college students with financial difficulties. A total of 547 eligible participants were assessed using the Core Self-Evaluations Scale, Self-Acceptance Questionnaire, and Interaction Anxiousness Scale. Correlation and mediation analyses revealed that core self-evaluations were significantly negatively correlated with social anxiety (*r* = −0.533, *p* < 0.001). Self-acceptance was positively correlated with core self-evaluations (*r* = 0.691, *p* < 0.001) and negatively correlated with social anxiety (*r* = −0.676, *p* < 0.001). The mediation analysis demonstrated that self-acceptance partially mediated the relationship between core self-evaluations and social anxiety. These findings suggest that self-acceptance serves as a partial mediator between core self-evaluations and social anxiety among economically disadvantaged college students. The results provide empirical evidence for developing mental health intervention programs in higher education settings, particularly highlighting the potential of enhancing self-acceptance as an effective approach to alleviate social anxiety in this population.

## 1 Introduction

In recent years, the mental health of impoverished college students has become a significant research topic in higher education (Li and Zhou, 2015). College students with financial difficulties have become a key concern group for mental health education in universities. Compared with non-impoverished students, they are more prone to varying degrees of psychological issues under the influence of multiple pressures such as economic, family, and academic stress. These problems include academic difficulties, interpersonal avoidance, emotional distress, and imbalanced personality development (Dong et al., 2020). College students with financial difficulties are on the verge of transitioning into the workforce, necessitating competent interpersonal skills. If their social anxiety is not properly alleviated and managed through appropriate interventions, they may struggle to adapt to social relationships and situations in society. This could eventually develop into social dysfunction, and have profoundly negative impacts on their future mental health, physical wellbeing, and career achievements (Zhang, 2016; Guo, 2020). Therefore, investigating the influencing factors and underlying mechanisms of social anxiety among college students with financial difficulties, as well as promoting preventive and intervention measures to safeguard their psychological and physical wellbeing, has become an urgent issue for mental health education in universities.

This study aimed to investigate whether self-acceptance mediates the relationship between core self-evaluations and social anxiety among college students with financial difficulties. Although existing research had accumulated some preliminary findings on social anxiety among this population, empirical studies remain limited. Furthermore, the precise factors and mechanisms contributing to their social anxiety were not yet well understood. By investigating self-acceptance as a mediator in the relationship between core self-evaluations and social anxiety, this research sought to elucidate the tripartite relationship, enrich the existing literature, provide novel insights for developing targeted interventions and treatments and offer practical implications for fostering better mental health outcomes in this vulnerable student population.

## 2 Literature review

### 2.1 Research status of core self-evaluation, social anxiety, and self-acceptance

According to Ministry of Education and Ministry of Finance of the People's Republic of China (2007), college students with financial difficulties refer to those “whose personal and family financial resources are insufficient to cover the basic costs of tuition and essential living expenses during their academic studies.” The classification system for these students adopts a three-tier structure: moderately disadvantaged, disadvantaged, and severely disadvantaged, with differentiated criteria applied to each category (Fujian Provincial Department of Education, 2023). The criteria for identifying students with special financial difficulties include: (1) recipients of special hardship support, or students from families receiving subsistence allowances or at the marginal level of such allowances; (2) orphans, HIV-infected students, and de facto parentless students; (3) students with disabilities; (4) children of martyrs, role models, military personnel, police officers, or comprehensive fire rescue team members who died or were disabled (grade 1–4) in the line of duty; and (5) children of entitled beneficiaries. Students from financially disadvantaged families are defined as those with disabled parents. General financial difficulty refers to students whose family economic status falls between disadvantaged and non-disadvantaged, bordering on the threshold of financial hardship. In actual assessment process, the final classification is determined through comprehensive evaluation of students' authentic financial hardships, which may result from medical expenses, natural disasters, or other adversities affecting either the students themselves or their cohabiting family members.

Social anxiety is one of the most common forms of anxiety (Jarcho et al., 2015; Fernández et al., 2018). It refers to the individual's expected negative evaluation of real or imagined social situations, as well as excessive expectations of making a good impression on others. When this expectation is beyond the scope of one's ability, it causes extreme uneasiness, accompanied by physiological reactions such as nervousness, blushing, and difficulty in expression. It also produces thoughts of wanting to escape (Purdon et al., 2001). This represents a prevalent form of social dysfunction among economically disadvantaged college students. Due to financial constraints, these students frequently exhibit low self-esteem, social isolation, and elevated anxiety in interpersonal contexts. Characteristically, they adopt self-isolating and marginalizing behaviors as maladaptive coping mechanisms to avoid social engagement (Luo et al., 2009; Guha, 2014). Drawing upon Social Exchange Theory (Homans, 1958), college students with financial difficulties tend to perceive their social resource endowment as a form of “disadvantage” and anticipate it may evolve into “negative capital” that could yield adverse outcomes. This cognitive appraisal heightens their sensitivity to negative evaluations in social interactions, consequently predisposing them to adopt avoidance strategies such as social withdrawal to minimize interpersonal engagement (Hofmann and DiBartolo, 2010). From the perspective of Erikson's psychosocial development theory (Erikson, 1968), the university period represents a critical transitional phase from adolescence to early adulthood, during which the primary developmental task involves establishing meaningful friendships while overcoming feelings of isolation. A multinational study spanning seven countries showed that the proportion of young people suffering from social anxiety ranges from 23% to 58% (Jefferies, 2020), while 87.8% of Chinese college students experience varying degrees of social anxiety (Stein et al., 2017; Wu et al., 2021). National statistics indicated that approximately 20% of Chinese university students come from economically disadvantaged families (Guo, 2020). These demographic faces unique psychosocial developmental dilemmas: while possessing strong innate needs for social connection, they simultaneously experience significant interpersonal conflicts due to resource deprivation stemming from financial constraints. This fundamental tension between social motivation and structural limitation predisposes this population to heightened vulnerability to social anxiety disorders. Further research indicated that the severity of social anxiety among college students with financial difficulties is significantly correlated with psychological resilience, particularly self-acceptance capacity (Neff, 2003). As a critical psychological resource, self-acceptance effectively buffers social stress and regulates anxiety responses. This empirical finding provides both theoretical justification and practical avenues for starting from the perspective of interpersonal relationship intervention aimed at enhancing the social adaptation capabilities of this student population.

Self-acceptance refers to an individual's positive attitude toward themselves and their inherent characteristics (Zheng, 2017), representing an emotional and attitudinal embrace of their actual self (Zhang, 2016). It plays a crucial role in healthy psychological development (Cong and Gao, 1999). Individuals with high self-acceptance tend to exhibit stronger cooperative abilities and better interpersonal skills (Ryan and Deci, 2000). Among college students, the level of self-acceptance are positively correlated with the quality of interpersonal relationships-the higher the self-acceptance, the more harmonious an individual's social interactions tend to be (Hu, 2011). Furthermore, self-acceptance negatively predicts negative emotional states such as anxiety and depression (Cunha and Paiva, 2012), and shows a strong positive correlation with self-esteem (Macinnes, 2010; Zhang et al., 2019). Core self-evaluation is a new approach to personality research (Liu, 2023) that involves the most basic evaluation and overall estimation of an individual's ability and value and thus forms the basis for other evaluations related to specific situations (Judge and Bono, 2001) and includes four core traits: self-esteem, locus of control, neuroticism, and self-efficacy (Judge et al., 1997; Du et al., 2007; Chen and Xiong, 2023). It serves as the most generalized self-perception (Judge et al., 2003). Studies indicate that individuals with high core self-evaluations experience less stress and tension, as well as fewer negative emotions such as fear and anxiety (Brunborg, 2008; Kammeyer-Mueller et al., 2009), compared to those with low core self-evaluation (Zhou et al., 2017). It can thus be inferred that there is a positive correlation between self-acceptance and core self-evaluation.

### 2.2 Mediating effect of self-acceptance

The cognitive model of social anxiety suggests that social anxiety originates from the fear of social evaluation and negative self-evaluation in anticipation of social dilemmas (Bautista and Hope, 2015). Social anxiety is thus closely related to self-evaluation. Individuals with low self-evaluation tend to be unable to view themselves correctly and find that it is not easy to engage in positive social interactions with others. This results in avoidance and social anxiety (Tian, 2007). Research has indicated that core self-evaluation can directly and negatively predict social anxiety: the lower the level of core self-evaluation, the higher the level of social anxiety tends to be (Hong and Song, 2014; Wen et al., 2016). Core self-evaluation can also have an indirect impact on social anxiety through factors such as sense of security, self-differentiation, and self-acceptance (Li and Meng, 2018; Yang, 2019; Wang et al., 2023). We therefore hypothesized that self-acceptance mediates the relationship between core self-evaluation and social anxiety among impoverished college students.

### 2.3 Conceptual framework and assumptions

In the theoretical model guiding the present study, the dependent variable was the social anxiety of impoverished college students, core self-evaluation was the independent variable, and self-acceptance was the mediating variable.

**Figure 1:**
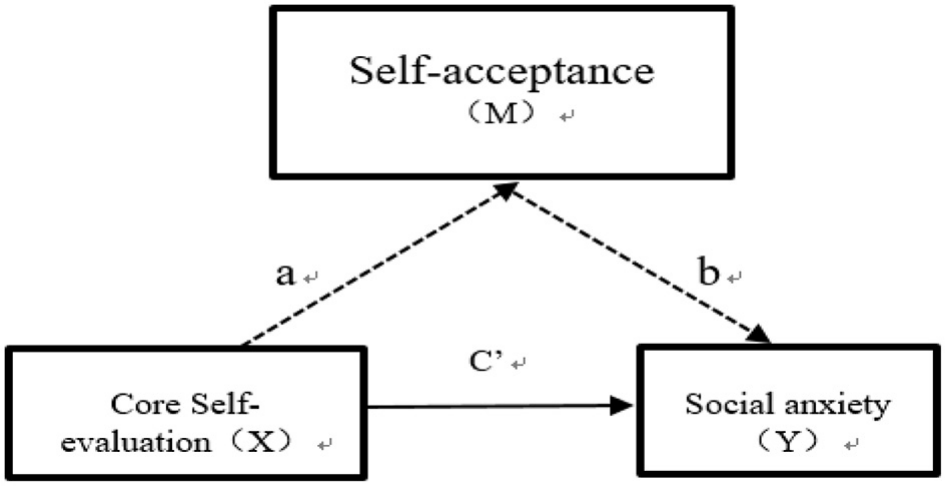
Theoretical model of self-acceptance.

Based on the conceptual framework shown in Figure 1, the following hypothesizes were proposed:

H1: The core self-evaluation of college students with financial difficulties is significantly negatively correlated with social anxiety;
H2: There is a significant negative correlation between self-acceptance and social anxiety among college students with financial difficulties;
H3: Self-acceptance mediates the relationship between core self-evaluation and social anxiety among college students with financial difficulties.

## 3 Methodology

### 3.1 Participants

The participants in this study were chosen through random sampling and comprised 547 impoverished college students, including students majoring in the natural sciences, humanities, and interdisciplinary studies, thus ensuring a broad representation of academic prospects. Participants completed the questionnaire in July 2024 under the supervision of the researchers. A total of 547 valid questionnaires were collected, comprising 234 male participants (42.8%) and 313 female participants (57.2%). The sample distribution across academic years was as follows: 130 freshmen (23.8%), 190 sophomores (34.7%), 166 juniors (30.3%), and 61 seniors (11.2%). Regarding geographical background, 249 participants (45.5%) came from urban areas while 298 (54.5%) were from rural regions. All participants were selected from the financial aid registry of college students with financial difficulties, which was established under the Fujian Province Identification Measures for Economically Disadvantaged Students (Fujian Provincial Department of Education, 2023), with prior informed consent obtained from each participant.

### 3.2 Measures

#### 3.2.1 Core self-evaluation scales

The Chinese version of the core self-evaluation scales (CSES) revised by (Du et al. 2012) was used. This is a one-dimensional self-assessment scales, contains 10 items. The items are scored on a 5-point Likert scale ranging from complete disagreement (1) to complete agreement (5). Six items (2, 3, 5, 7, 8, and 10) are reverse scored. Higher scores indicate a more positive core self-evaluation. The Cronbach's alpha coefficient for the scale in this study was 0.83. The Cronbach's alpha coefficient for this scale in the present study was 0.89.

#### 3.2.2 Interaction anxiousness scale

The interaction anxiousness scale (IAS) developed by Leary in 1983 and revised by Peng et al. (2004) was adopted in this study. This scale contains 15 items, which are scored on a 5-point Likert scale ranging from completely inconsistent (1) to completely consistent (5). Four items (3, 6, 10, and 15) are reverse scored. Higher scores indicate that individuals are more likely to experience anxiety. The internal alpha coefficient of the scale was 0.81, and the test-retest coefficient was 0.78. The internal consistency of the entire scale was 0.86.

#### 3.2.3 Self-acceptance questionnaire

The self-acceptance questionnaire (SAQ) based on the scale developed by Cong and Gao (1999) was used. This scale contains 16 items divided into two factors: self-acceptance and self-evaluation. The items are scored on a 4-point Likert scale ranging from very different (1) to very similar (4). Eight items (1, 4, 7, 8, 11, 13, 14, and 16) are reverse scored. A higher score indicates a higher level of self-acceptance. The internal alpha coefficient of the questionnaire was 0.86, and the test-retest reliability was 0.77. The internal consistency across the entire scale was 0.88.

### 3.3 Data processing and analysis

Statistical analysis was conducted using SPSS 26.0 and the PROCESS v. 4.2. First, common method bias assessed using exploratory factor analysis. Means and standard deviations were determined for each variable, along with Pearson correlation analyses. The bias-corrected non-parametric percentile bootstrap method was then applied to evaluate the mediated effects (Wen and Ye, 2014). Hayes's PROCESS macro model 6 was adopted to estimate mediation effects; this involved a resampling process with 5,000 iterations to establish a 95% confidence interval.

## 4 Results

### 4.1 Common method bias

Exploratory factor analysis was adopted to assess potential common method bias (Zhou and Long, 2004), and the results showed that there were seven factors with eigenvalues greater than 1. The rate of variance explained by the first factor was 30.1%, which is less than the critical standard of 40%. This indicated that common method bias was not a problem in this study.

### 4.2 Descriptive statistics

The descriptive statistics of all variables are presented in Table 1. Comparative analysis was conducted based on the results for core self-evaluation, self-acceptance, and social anxiety in this study and the results of previous studies. In the present study, the core self-evaluation scores (M = 35.19, SD = 5.95) of impoverished college students were significantly lower than the sample mean 37.5 in the study by Du et al. (2012; *t* = −7.300, *p* < 0.001), which indicates relatively low levels of core self-evaluation in the current cohort. The self-acceptance score (M = 40.66, SD = 6.39) was also significantly lower than the sample mean of 42.06 points in the study by Cong and Gao (1999; *t* = −5.115, *p* < 0.001), which indicates a generally low level of self-acceptance among impoverished college students. The social anxiety score (M = 46.62, SD = 8.53) was significantly higher than the sample mean of 38.78 points in the study by Peng et al. (2004; *t* = 21.492, *p* < 0.001), which indicates a relatively high level of social anxiety among the current cohort of impoverished college students.

**Table 1:** T-test: core self-evaluations, self-acceptance, and social anxiety compared to norms.

**Variables**	**M**	**SD**	**Norm M**	**Norm SD**	***t***
Core self-evaluation	35.19	5.95	37.05	5.50	−7.300^***^
Self-acceptance	46.62	8.53	38.78	8.50	21.492^***^
Social anxiety	40.66	6.39	42.06	6.63	−5.115^***^

*n* = 547; ^***^p < 0.001.

### 4.3 Correlation analysis

The results of the correlation analysis for core self-evaluation, self-acceptance, and social anxiety are shown in Table 2. Social anxiety was significantly negatively correlated with both core self-evaluation (*r* = −0.533, *p* < 0.001) and self-acceptance (*r* = −0.691, *p* < 0.001). Self-acceptance was significantly positively correlated with core self-evaluation (*r* = 0.691, *p* < 0.001). It thus appears there are close relationships among core self-evaluation, self-acceptance, and social anxiety, with pairwise correlations.

**Table 2:** Correlation analysis: core self-evaluation, self-acceptance, and social anxiety.

**Variables**	**M**	**SD**	**1**	**2**	**3**
Core self-evaluation	35.19	5.95	1		
Self-acceptance	40.66	6.39	0.691^**^	1	
Social anxiety	46.62	8.53	−0.533^**^	−0.676^**^	1

^*^p < 0.05, ^**^p < 0.01.

### 4.4 Mediating effects

**Figure 2:**
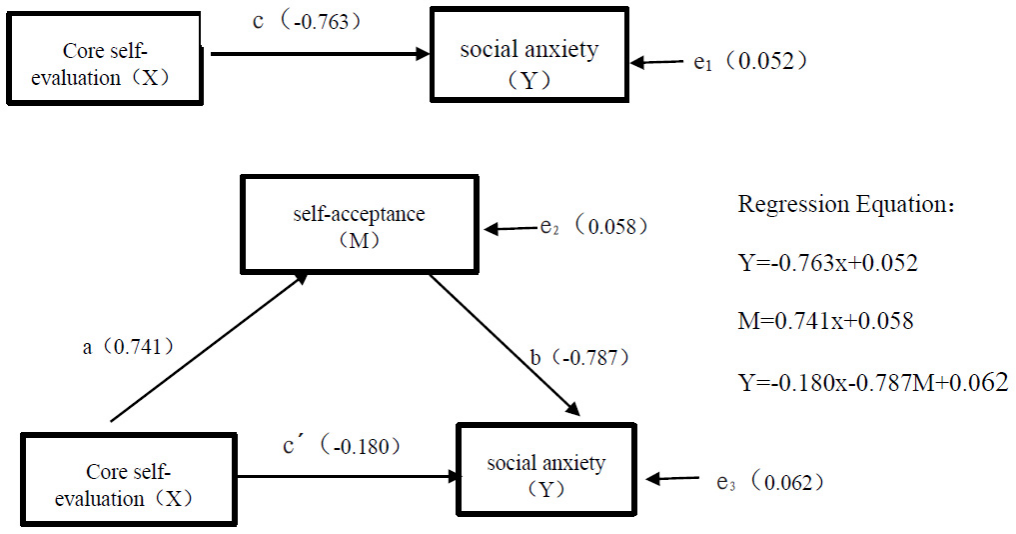
Self-acceptance meditation effect model.

The correlation analysis results indicated that there were significant pairwise correlations between core self-evaluation, self-acceptance, and social anxiety among college students with financial difficulties. Therefore, the mediating effect of self-acceptance can be further examined (Wen et al., 2004). The bias corrected non-parametric percentile Bootstrap method in Hayes's PROCESS macro-model 6 was used to test for the mediating effect, and the model was resampled 5,000 times to test the model fit and the significance of each path coefficient (Hayes, 2012). The results of the sequential tests are shown in Table 3. When core self-evaluation was taken as the independent variable for regression analysis, the regression coefficient *c* was −0.763, and the test result is significant (*t* = −14.687, *p* < 0.001). Core self-evaluation thus significantly and positively predicts self-acceptance. Regression coefficient *a* was 0.741, and the test result was significant (*t* = 22.329, *p* < 0.001), so self-acceptance significantly and negatively predicts social anxiety. Regression coefficient *b* was −0.787, and the test result was significant (*t* = −13.564, *p* < 0.001). With social anxiety as the dependent variable, and core self-evaluation and self-acceptance as independent variables, the regression coefficient *c'* was −0.180, and the test result was significant (*t* = −2.891, *p* < 0.01). The standardized regression coefficients *c, a, b*, and *c'* all reached the level of significance. Additionally, the mediation effect value for college students with financial difficulties was −0.583, accounting for 76.4% of the total effect. Specific results are presented in Table 4, indicating that self-acceptance thus partially mediated the relationship between the core self-evaluation and social anxiety of impoverished college students. The path diagram of the mediating effect is shown in Figure 2.

**Table 3:** Mediating effects of core self-evaluation and self-acceptance on social anxiety.

**Independent variable**	**Dependent variable**	***R***	***R^2^***	**ß**	**LLCI**	**ULCI**	***t***
Core self-evaluation	Social anxiety	0.533	0.284	−0.763	−0.866	−0.661	−14.687^***^
Core self-evaluation	Self-acceptance	0.691	0.478	0.741	0.676	0.807	22.329^***^
Core self-evaluation	Social anxiety	0.682	0.465	−0.18	−0.302	−0.058	−2.891^**^
Self-acceptance				−0.787	−0.901	−0.673	−13.564^***^

^**^p < 0.01,^***^p < 0.001.

**Table 4:** Paths, effect sizes, and proportions of variables on social anxiety in the model.

**Path**	**Standardized effect size**	**Effect proportion**	**95% confidence interval (Lower, Upper)**
Direct effect: Core self-evaluation → Social anxiety	−0.180	23.60%	[−0.302, −0.058]
Indirect effect: Core self-evaluation → Self-acceptance → Social anxiety	−0.583	76.40%	[−0.609, −0.543]
Total effect	−0.763	—	[−0.866, −0.661]

## 5 Discussion

### 5.1 Differential characteristics

Departing from prior research on general college student populations, this study specifically targets college students with financial difficulties to analyze how core self-evaluation and self-acceptance influence social anxiety. The findings demonstrate that the core self-evaluation of college students with financial difficulties was significantly lower than the norm, which is consistent with the findings of Liu and Jiang (2019). The self-acceptance of impoverished college students was significantly lower than the norm. The social anxiety level of college students with financial difficulties was significantly lower than the norm, consistent with the findings of Guo (2020). Thus, college students with financial difficulties demonstrated relatively lower levels of core self-evaluation and self-acceptance. They tend to be more introverted and prone to psychological problems such as low self-esteem and timidity. In social situations, they are also likely to exhibit negative behaviors such as avoidance and withdrawal, as well as being highly likely to experience negative emotions like anxiety and depression. All these greatly reduce the willingness of these students to communicate with others. College students with financial difficulties thus appear to experience more psychological pressure due to their economic hardships. These economic challenges are detrimental to the all-around development of these students, who are influenced by various factors such as social comparison, differences in cultural capital, and employment pressure. These students were prone to doubting their own abilities, which in turn affects their self-confidence and sense of self-worth. This low sense of self-efficacy further reduces their self-evaluation (Zhou et al., 2017).

### 5.2 Effect of core self-evaluation on social anxiety

The findings indicated that there was a significant negative correlation between core self-evaluation and social anxiety among impoverished college students: the higher the core self-evaluation, the lower the level of social anxiety among these students tended to be. Self-evaluation plays a crucial role in the formation of social anxiety (Guo, 2020). This result appeared that the level of core self-evaluation affects the levels of social anxiety among impoverished college students (Wen et al., 2016; Liu and Jiang, 2019). Individuals with a high level of core self-evaluation tend to have a more positive assessment of their own abilities and values; they experience more positive emotions and possess greater self-confidence. Impoverished college students with high core self-evaluation enjoy better mental health, as this high self-assessment is beneficial to their psychological wellbeing. They also have relatively higher satisfaction with life and work (Judge, 2009). In social situations, they were more likely to hold a positive attitude and show a greater willingness to communicate, which results in a lower level of social anxiety. Furthermore, students with lower core self-evaluation consider themselves to be “of low value” and are more prone to negative emotions such as anxiety and depression. Excessive negative emotional experiences increase the likelihood of triggering psychological problems in this population (Lei et al., 2018).

### 5.3 Effect of self-acceptance on social anxiety

This study revealed that there was a significant negative correlation between self-acceptance and social anxiety among college students with financial difficulties, which is consistent with previous research findings—namely that the higher the level of self-acceptance, the lower the level of social anxiety is likely to be (Zhang, 2016; Wang et al., 2023; Leary et al., 2007). The negative emotional reactions shown by an individual in social situations are closely related to the individual's level of self-acceptance (Wang, 2021). Individuals with a high level of self-acceptance tend to have an objective understanding of themselves (Neff, 2003), and they can present themselves appropriately, rationally control their emotions (Gross, 2010), and appropriately regulate their level of social anxiety. The current findings indicate that individuals with a high level of social anxiety not only worry about negative evaluations from others in social situations, but even positive evaluations can trigger anxiety (Reichenberger and Blechert, 2018). Such individuals do not want their good performance to draw attention, nor do they wish for others' expectations of them to increase as a result (Weeks and Zoccola, 2016; Alden et al., 2008). Individuals with a low level of self-acceptance are thus prone to feeling that others look down on them, or they may magnify minor mistakes in social interactions. They are thus more likely to exhibit social behaviors of rejection and avoidance in social situations (Kong and Zhang, 2007; Weeks and Zoccola, 2016).

### 5.4 Mediating role of self-acceptance

The model indicated that core self-evaluations in college students with financial difficulties directly and negatively predict social anxiety, suggesting that core self-evaluations play a significant role in influencing social anxiety (Judge, 2009; Orth and Robins, 2022). Individuals with higher core self-evaluations tend to maintain a more positive cognitive orientation toward their own abilities and self-worth. This stable sense of self-value enables them to exhibit dual adaptive characteristics in social interactions. On one hand, they are better able to maintain an objective assessment of social situations; on the other hand, they can effectively regulate negative information encountered in social contexts and are less likely to attribute such negative feedback to themselves. Consequently, these individuals exhibit lower levels of social anxiety (Rapee and Heimberg, 1997).

The model revealed that core self-evaluations positively predict self-acceptance among college students with financial difficulties (Judge et al., 2005), indicating that those with higher core self-evaluations tend to exhibit greater self-acceptance. Furthermore, the model demonstrated that core self-evaluations primarily influence social anxiety through the mediating role of self-acceptance, accounting for 76.4% of the total mediating effect. The findings suggested that, in social contexts, college students with financial difficulties with lower core self-evaluations were more prone to negative emotions such as social anxiety due to their lack of self-acceptance regarding their social performance (Heimberg et al., 2014). Specifically, these students often hold negative appraisals of their own social competence. When faced with social interactions, they were more likely to focus on their perceived shortcomings (Spurr and Stopa, 2002), triggering self-doubt and emotional distress. This self-critical cognitive pattern further reinforces their social avoidance behaviors and anxiety experiences, creating a vicious cycle.

This study constructed a mediation model to examine the process and mechanism through which core self-evaluations influence social anxiety among college students with financial difficulties. The results confirmed the mediating role of self-acceptance in the relationship between core self-evaluations and social anxiety, validating the proposed mediation model: core self-evaluations → self-acceptance → social anxiety. The findings partially support the core assumption of interpersonal theory, which posits that individuals' negative self-evaluations (low core self-evaluations) may impair self-acceptance, thereby contributing to social anxiety. This discovery not only provides empirical support for interpersonal theory but also offers a novel theoretical perspective for understanding the formation of social anxiety among college students with financial difficulties. Furthermore, it holds significant practical implications for designing targeted psychological interventions.

Notwithstanding its contributions, this study had several limitations that warrant consideration. First, the cross-sectional design precludes causal inferences regarding the relationships among core self-evaluation, self-acceptance, and social anxiety in college students with financial difficulties. Second, reliance on self-report measures may introduce biases such as social desirability effects. Third, the sample was restricted to students from a single geographic region, potentially limiting the generalizability of findings due to homogeneous cultural and socioeconomic characteristics. Finally, while the study identified self-acceptance as a mediator, other salient factors (e.g., social support, self-esteem, psychological resilience) that may influence social anxiety were not examined. Future research should incorporate these variables to elucidate the complex mechanisms underlying social anxiety in this population, enhance the explanatory power of the mediation model, and provide novel insights for developing targeted interventions to improve their psychosocial wellbeing.

### 5.5 Significance

This study explored the potential factors influencing the relationship between core self-evaluation on social anxiety among college students with financial difficulties. This study also explored the mediating role of self-acceptance in this relationship. This makes a theoretical contribution to the study of college students' social anxiety, particularly for the population of Chinese college students with financial difficulties. In following up on the theoretical contributions and applying them to practice, Chinese education managers could pursue interventions targeting the social anxiety of college students with financial difficulties by cultivating their core self-evaluation to improve self-acceptance and alleviate social anxiety.

## 6 Conclusions, limitations, and future directions

### 6.1 Conclusions

Core self-evaluation, self-acceptance, and social anxiety were negatively correlated among impoverished college students, while self-acceptance and core self-evaluation were positively correlated. Self-acceptance partially mediated the relationship between core self-evaluation and social anxiety. These findings provided a theoretical basis for interventions to prevent or reduce social anxiety in this population. Such interventions could be carried out by reducing these students' negative thoughts about themselves, others, and the outside world while enhancing their core self-evaluation levels and decreasing social avoidance and withdrawal behaviors.

### 6.2 Limitations and future directions

Due to the restrictions of time, funds, and resources, this study has some limitations. First, data collection completely depended on self-report tools, and the reliability of the results may have been affected by social expectations. Second, all participants in this study were undergraduate students recruited from a single university. This sampling characteristic may limit the generalizability of our findings across different age groups and cultural contexts. Consequently, the current results should be interpreted as most applicable to economically disadvantaged college students within East Asian cultural settings. Finally, while our sample size (*N* = 547) meets basic statistical requirements, future studies should incorporate additional variables closely related to social anxiety (e.g., self-esteem, social support) to better understand the underlying mechanisms and improve the mediation model's explanatory power regarding economically disadvantaged undergraduates' mental health.

## Data Availability

The raw data supporting the conclusions of this article will be made available by the authors, without undue reservation.

## References

[B1] Alden L. E. Taylor C. T. Mellings T. M. Laposa J. M. (2008). Social anxiety and the interpretation of positive social events. J. Anxiety Disord. 22, 577–590. doi: 10.1016/j.janxdis.05.007 17587542

[B2] Bautista L. C. Hope A. D. (2015). Fear of negative evaluation, social anxiety and response to positive and negative online social cues. Cognit. Ther. Res. 39, 658–668. doi: 10.1007/s10608-015-9687-3

[B3] Brunborg G. S. (2008). Core self-evaluations: a predictor variable for job stress. Eur. Psychol. 13, 96–102. doi: 10.1027/1016-9040.13.2.96

[B4] Chen J. Xiong M. (2023). Effects of relative deprivation on college students' social anxiety: a moderated mediating model. Stud. Psychol. Behav. 21, 65–71. doi: 10.12139/j.1672-0628.01.010

[B5] Cong Z. Gao W. F. (1999). A preliminary study on the correlation between self-acceptance and social avoidance and distress in college students. Chin. J. Behav. Med. Sci. 8, 119–120.

[B6] Cunha M. Paiva M. J. (2012). Text anxiety in adolescents: the role of self-criticism and acceptance and mindfulness skills. Span. J. Psychol. 15, 533–543. doi: 10.5209/rev_sjop.2012.v15.n2.38864 22774427

[B7] Dong X. Chen Q. M. Hu Y. H. Lu Y. C. (2020). Study on the mental health status and intervention of college students with financial difficulties. Educ. Reform Dev. 2, 22–25. doi: 10.26689/ERD.V2I1.1324

[B8] Du J. Z. Zhang X. Zhao Y. (2007). Reliability, validation and construct confirmatory of core self-evaluations scale. Psychol. Res. 15, 116–121.

[B9] Du J. Z. Zhang X. Zhao Y. (2012). Reliability, validation and construct confirmatory of core self-evaluations scale. Psychol. Res. 5, 54–60.

[B10] Erikson E. H. (1968). Identity: Youth and Crisis. New York: Norton.

[B11] Fernández R. S. Pedreira M. E. Boccia M. M. Kaczer L. (2018). Commentary: forgetting the best when predicting the worst: preliminary observations on neural circuit function in adolescent social anxiety. Front. Psychol. 9:1088. doi: 10.3389/fpsyg.2018.01088 30050477 PMC6051016

[B12] Fujian Provincial Department of Education (2023). Implementation measures for identifying financially disadvantaged students in Fujian Province (Min Jiao Gui [2023] No. 1).

[B13] Gross J. (2010). Emotion regulation: affective, cognitive, and social consequences. Psychophysiology 39, 281–291. doi: 10.1017/S0048577201393198 12212647

[B14] Guha M. (2014). Diagnostic and Statistical Manual of Mental Disorders: DSM-5 (5th edition). Ref. Rev. 28, 36–37. doi: 10.1108/RR-10-2013-0256

[B15] Guo W. (2020). The relationship between perceived social support and social anxiety among impoverished college students (Master's thesis). Minnan Normal University, Zhangzhou, China. doi: 10.27726/d.cnki.gzzsf.2020.000152

[B16] Hayes A. F. (2012). PROCESS: A Versatile Computational Tool for Mediated Moderation, Moderation, and Conditional Process Analysis. Guilford Press, 1–39.

[B17] Heimberg R. G. Brozovich F. A. Rapee R. M. (2014). “A cognitive-behavioral model of social anxiety disorder,” in Social Anxiety : Clinical, Developmental, and Social Perspectives, eds. S. G. Hofmann and P. M. DiBartolo, 3rd Edn. (Lodnon: Academic Press), 705–728.

[B18] Hofmann S. G. DiBartolo P. M. (Eds.). (2010). Social Anxiety: Clinical, Developmental, and Social Perspectives, 2nd Edn. Cambridge, MA: Academic Press.

[B19] Homans G. C. (1958). Social behavior as exchange. Am. J. Sociol. 63, 597–606. doi: 10.1086/222355

[B20] Hong Y. J. Song X. C. (2014). The moderating and mediating effects of self-esteem in the relationship between self-esteem need and social distress. Chin. J. Health Psychol. 22, 285–287. doi: 10.13342/j.cnki.cjhp.02.052

[B21] Hu Q. H. (2011). The relationship between acceptance, interpersonal relationship and social anxiety of undergraduates. [Master's Thesis]. Guangxi Normal University, Guilin, China.

[B22] Jarcho J. M. Romer A. L. Shechner T. Galvan A. Guyer A. E. Leibenluft E. . (2015). (2015). Forgetting the best when predicting the worst: preliminary observations on neural circuit function in adolescent social anxiety. Dev. Cogn. Neurosci. 13, 21–28. doi: 10.1016/j.dcn.03.002 25933410 PMC4466042

[B23] Jefferies P. M. (2020). Social anxiety in young people: a prevalence study in seven countries. PLoS ONE. 15:e0239133. doi: 10.1371/journal.pone.0239133 32941482 PMC7498107

[B24] Judge A. T. (2009). Core self-evaluations and work success. Curr. Dir. Psychol. Sci. 18, 58–62. doi: 10.1111/j.1467-8721.2009.01606.x

[B25] Judge T. A. Bono J. E. (2001). Relationship of core self-evaluations traits–self-esteem, generalized self-efficacy, locus of control, and emotional stability–with job satisfaction and job performance: a meta-analysis. J. Appl. Psychol. 86, 80–92. doi: 10.1037/0021-9010.86.1.80 11302235

[B26] Judge T. A. Bono J. E. Erez A. Locke E. A. (2005). Core self-evaluations and job and life satisfaction: the role of self-concordance and goal attainment. J. Appl. Pychol. 90, 257–68. doi: 10.1037/0021-9010.90.2.257 15769236

[B27] Judge T. A. Erez A. Bono J. E. Thoresen C. J. (2003). The coreself-evaluations scale: development of a measure. Personnel Psychol. 56, 303–331. doi: 10.1111/j.1744-6570.2003.tb00152.x

[B28] Judge T. A. Locke E. A. Durham C. C. (1997). The dispositional causes of job satisfaction: a core evaluations approach. Res. Organ. Behav. 19, 151–188.

[B29] Kammeyer-Mueller J. D. Judge T. A. Scott B. A. (2009). The role of core self-evaluations in the coping process. J. Appl. Psychol. 94, 177–95. doi: 10.1037/a0013214 19186903

[B30] Kong D. S. Zhang W. (2007). Relationship between life events, way of coping, social support and subjective well-being of impoverished college students. Chin. J. Clin. Psychol. 1, 61–62+65. doi: 10.16128/j.cnki.1005-3611.2007.01.024

[B31] Leary M. R. Tate E. B. Adams C. E. Allen A. B. Hancock J. (2007). Self-compassion and reactions to unpleasant self-relevant events: the implications of treating oneself kindly. J. Pers. Soc. Psychol. 92, 887–904. doi: 10.1037/0022-3514.92.5.887 17484611

[B32] Lei X. Wang J. Zhang Y. Ye B. Liu C. (2018). Core self-evaluation and depression: a chain mediating model. Chin. J. Clin. Psychol. 26, 808–810+830. doi: 10.16128/j.cnki.1005-3611.04.039

[B33] Li F. L. Zhou C. X. (2015). Characteristics and trend of college students' mental health education research in China—based on the bibliometric analysis. J. Southwest Univ. 41, 102–107+207. doi: 10.13718/j.cnki.xdsk.05.014

[B34] Li X. J. Meng Y. F. (2018). The influence of college students' core self-evaluation on social anxiety-from the perspective of self-differentiation. J. Fujian Med. Univ. 19, 29–32+2.

[B35] Liu T. Jiang F. (2019). A survey on the relationship between core self-evaluations and mental health level for college students with financial difficulties. J. Yueyang Vocat. Tech. Coll. 34, 49–53. doi: 10.13947/j.cnki.yyzyxb.02.012

[B36] Liu Y. (2023). A literature review of core self-evaluation of college students in China. J. Chongqing Univ. Educ. 36, 117–122.

[B37] Luo F. S. Shen D. Zhang S. M. Wang X. F. Yuan H. M. Li Z. Q. (2009). Mental health status of poverty students and its influencing factors. Chin. J. Clin. Psychol. 17, 272–274. doi: 10.16128/j.cnki.1005-3611.03.037

[B38] Macinnes D. L. (2010). Self-esteem and self-acceptance: an examination into their relationship and their effect on psychological health. J. Psychiatr. Ment. Health Nurs. 13, 483–489. doi: 10.1111/j.1365-2850.2006.00959.x 16965465

[B39] Ministry of Education and Ministry of Finance of the People's Republic of China. (2007). Guanyu renzhen zuohao gaodeng xuexiao jiating jingji kunnan xuesheng rending gongzuo de zhidao yijian [Guidance on the diligent implementation of the identification of students from economically disadvantaged families in higher education institutions] (Document No. 教财 [2007]8号). Available online at: http://www.moe.gov.cn/srcsite/A05/s7052/200706/t20070626_181382.html

[B40] Neff K. D. (2003). Self-compassion: an alternative conceptualization of a healthy attitude toward oneself. Self Identity 2, 85–101. doi: 10.1080/15298860309032

[B41] Orth U. Robins R. W. (2022). Is high self-esteem beneficial? Revisiting a classic question. Am. Psychol. 77, 5–17. doi: 10.1037/AMP0000922 35357851 PMC9306298

[B42] Peng C. Z. Gong Y. X. Zhu X. Z. (2004). The applicabiliy of interaction anxiousness scale in Chinese undergraduate students. Chin. Ment. Health J. 27, 39–41. doi: 10.1007/BF02911031

[B43] Purdon C. Antony M. Monteiro S. Swinson R. P. (2001). Social anxiety in college students. J. Anxiety Disord. 15, 203–215. doi: 10.1016/S0887-6185(01)00059-7 11442139

[B44] Rapee R. M. Heimberg R. G. (1997). A cognitive-behavioral model of anxiety in social phobia - sciencedirect. Behav. Res. Ther. 35, 741–756. doi: 10.1016/S0005-7967(97)00022-3 9256517

[B45] Reichenberger J. Blechert J. (2018). Malaise with praise: a narrative review of 10 years of research on the concept of fear of positive evaluation in social anxiety. Depress Anxiety 35, 1228–1238. doi: 10.1002/da.22808 30144225 PMC6519229

[B46] Ryan R. M. Deci E. L. (2000). Self-determination theory and the facilitation of intrinsic motivation, social development, and well-being. Am. Psychol. 55, 68–78. doi: 10.1037/0003-066X.55.1.68 11392867

[B47] Spurr J. M. Stopa L. (2002). Self-focused attention in social phobia and social anxiety. Clin. Psychol. Rev. 22, 947–975. doi: 10.1016/S0272-7358(02)00107-1 12238248

[B48] Stein D. J. Lim C. C. W. Roest A. M. de Jonge P. Aguilar-Gaxiola S. Al-Hamzawi A. . (2017). The cross-national epidemiology of social anxiety disorder: data from the world mental health survey initiative. BMC Med. 15:143. doi: 10.1186/s12916-017-0889-2 28756776 PMC5535284

[B49] Tian X. Y. (2007). Relationship of emotion expression and personal evaluation with social anxiety among undergraduates. Chin. J. Sch. Health. 28, 991–992.

[B50] Wang Y. (2021). A study of the relationship between self-acceptance and social anxiety of college students (Master's thesis). Wuhan University, Wuhan, China. doi: 10.27379/d.cnki.gwhdu.2021.001560

[B51] Wang Y. H. Wang Z. Peng Y. C. (2023). The influence of college students' core self-evaluation on social anxiety: the mediating effect of self-acceptance. Psychol. Monthly. 18, 40–42+50.

[B52] Weeks J. W. Zoccola P. M. (2016). Fears of positive versus negative evaluation: distinct and conjoint neuroendocrine, emotional, and cardiovascular responses to social threat. J. Exp. Psychopathol. 7, 632–654. doi: 10.5127/jep.056016

[B53] Wen Y. Yang Y. J. Yue C. Z. (2016). An investigation into the relationship between social anxiety, core self-evaluation and core self-reflection in university students. Health Med. Res. Pract. 13, 14–17. doi: 10.11986/j.issn.1673-873X.02.004

[B54] Wen Z. L. Ye B. J. (2014). Analyses of mediating effects: the development of methods and models. Adv. Psychol. Sci. 22, 731–745. doi: 10.3724/SP.J.2014.00731

[B55] Wen Z. L. Zhang L. Hou J. T. Liu H. Y. (2004). Testing and application of the mediating effects. Acta Psychol. Sin. 36, 614–620.

[B56] Wu T. Yang N. X. Cai L. Zhang Y. Sang Z. Q. (2021). Self-compassion and social anxiety: the mediation effect of self-esteem and fear of evaluation. Chin. J. Clin. Psychol. 29, 169–172+78. doi: 10.16128/j.cnki.1005-3611.01.034

[B57] Yang H. M. (2019). A study on the relationship among mindfulness, core self-evaluation, sense of security and social anxiety of college students. [Master's thesis]. Harbin Engineering University Harbin, China.

[B58] Zhang Q. Zhang Y. W. Wu R. G. Hao S. W. Xu Z. L. Guan R. Y. . (2019). Dual mediating effect of self-efficacy and perceived social support on the relationship between self-acceptance and self-esteem in college students. Chin. J. Clin. Psychol. 27, 1879–1884. doi: 10.13342/j.cnki.cjhp.12.033

[B59] Zhang Z. X. (2016). A study on the Relationship between college students' self-acceptance and social anxiety: the mediating role of perceived social support. [Master's thesis]. Jilin University, Jilin, China.

[B60] Zheng H. Y. (2017). A review of research on self-acceptance. Modern Commun. 25, 25–26.

[B61] Zhou H. Long L. R. (2004). Statistical remedies for common method biases. Adv. Psychol. Sci. 12, 942–950.

[B62] Zhou Y. Chen J. Z. Zhang H. Wang P. Yang Y. L. Dong X. B. (2017). Influence of core self-evaluation on cognitive failure: mediation of boredom proneness. Chin. J. Clin. Psychol. 25, 132–137. doi: 10.16128/j.cnki.1005-3611.01.029

